# Effects of GABA Supplementation on Intestinal SIgA Secretion and Gut Microbiota in the Healthy and ETEC-Infected Weanling Piglets

**DOI:** 10.1155/2020/7368483

**Published:** 2020-05-26

**Authors:** Yuanyuan Zhao, Jing Wang, Hao Wang, Yonggang Huang, Ming Qi, Simeng Liao, Peng Bin, Yulong Yin

**Affiliations:** ^1^Guangdong Provincial Key Laboratory of Animal Nutrition Control, Institute of Subtropical Animal Nutrition and Feed, College of Animal Science, South China Agricultural University, Guangzhou, China; ^2^Hunan International Joint Laboratory of Animal Intestinal Ecology and Health, Laboratory of Animal Nutrition and Human Health, College of Life Sciences, Hunan Normal University, Changsha 410081, China; ^3^College of Animal Science and Technology, Hunan Agricultural University, Changsha, 410128 Hunan, China; ^4^Jiangsu Co-Innovation Center for Important Animal Infectious Diseases and Zoonoses, Joint International Research Laboratory of Agriculture and Agri-Product, Safety of Ministry of Education of China, College of Veterinary Medicine, Yangzhou University, Yangzhou, China

## Abstract

Pathogenic enterotoxigenic *Escherichia coli* (ETEC) has been considered a major cause of diarrhea which is a serious public health problem in humans and animals. This study was aimed at examining the effect of *γ*-aminobutyric acid (GABA) supplementation on intestinal secretory immunoglobulin A (SIgA) secretion and gut microbiota profile in healthy and ETEC-infected weaning piglets. A total of thirty-seven weaning piglets were randomly distributed into two groups fed with the basal diet or supplemented with 40 mg·kg^−1^ of GABA for three weeks, and some piglets were infected with ETEC at the last week. According to whether ETEC was inoculated or not, the experiment was divided into two stages (referred as CON1 and CON2 and GABA1 and GABA2). The growth performance, organ indices, amino acid levels, and biochemical parameters of serum, intestinal SIgA concentration, gut microbiota composition, and intestinal metabolites were analyzed at the end of each stage. We found that, in both the normal and ETEC-infected piglets, jejunal SIgA secretion and expression of some cytokines, such as IL-4, IL-13, and IL-17, were increased by GABA supplementation. Meanwhile, we observed that some low-abundance microbes, like *Enterococcus* and *Bacteroidetes*, were markedly increased in GABA-supplemented groups. KEGG enrichment analysis revealed that the nitrogen metabolism, sphingolipid signaling pathway, sphingolipid metabolism, and microbial metabolism in diverse environments were enriched in the GABA1 group. Further analysis revealed that alterations in microbial metabolism were closely correlated to changes in the abundances of *Enterococcus* and *Bacteroidetes*. In conclusion, GABA supplementation can enhance intestinal mucosal immunity by promoting jejunal SIgA secretion, which might be related with the T-cell-dependent pathway and altered gut microbiota structure and metabolism.

## 1. Introduction

Postweaning is a critical stage in swine husbandry, because inappropriate management procedures in this stage may cause health problems in the swine industry and lead to significant economic losses [1]. Weaning piglets are quite vulnerable to a variety of environmental stressors [2] and pathogens (e.g., enterotoxigenic *Escherichia coli* (ETEC)) [3], which could induce severe diarrhea and pose great threat to the health of weaning piglets. To deal with these problems, antibiotics have been used extensively to prevent pathogen infections. However, the widespread use of antibiotics in farm animals has been proved to cause severe problems like pathogenic drug resistance [4]. Thus, it is necessary to develop effective nutritional regulatory strategies to enhance intestinal immunity and prevent intestinal infection in weaning piglets.

Immunoglobulin A (IgA) secreted by plasma cells is the most abundant immunoglobulin in the body and is of critical importance in intestinal mucosal immunity [5]. The function of SIgA in intestinal mucosal immunity includes immune exclusion, antigen presentation, and interaction with gut commensals [6–10]. Therefore, it is clear that SIgA plays a critical role in maintaining intestinal mucosal immunity and preventing intestinal infection. A previous study reported that the fecal SIgA concentration in piglets reached the peak within a few days after birth [11]. After that, it constantly decreased to a relatively low level in about 10 days [11]. From then on, the fecal SIgA concentration in piglets remained low until at least 50 days of age [11]. This suggests that lack of SIgA might be an underlying reason why weaning piglets are so susceptible to numerous stressors and pathogens.

Current studies revealed that dietary amino acid supplementation, such as glutamine, arginine, and leucine, is an effective way to promote intestinal immunity and health [12–16]. Gamma-aminobutyric acid (GABA) is a well-known neurotransmitter generated through the decarboxylation of glutamic acid (Glu) catalyzed by glutamic acid decarboxylase (GAD) and also has critical roles in the immune system [17, 18]. Recent years have witnessed a growing interest in the application of GABA in animal husbandry. For instance, dietary GABA supplementation reduced the negative influences of weaning stress on weanling piglets by reducing aggressive behavior and regulating endocrine hormones [19]. For chicks under beak trimming stress, the supplementation of GABA significantly improves the immune response of chicks [20]. Our previous study also revealed that GABA supplementation can modulate the intestinal functions, including intestinal immunity, intestinal amino acid profiles, and gut microbiota in weanling piglets [21]. Furthermore, recent studies reported that GABA could alleviate intestinal pathogenic infection through attenuating epithelial cell apoptosis and promoting host Th17 responses [22, 23]. Moreover, a previous study found that intestinal microbiota-derived GABA also could increase intestinal IL-17 expression by activating mechanistic target of rapamycin complex 1- (mTORC1-) ribosomal protein S6 kinase 1 (S6K1) signaling in the context of ETEC or *Citrobacter rodentium* infection and drug-induced intestinal inflammation [24]. These studies have raised the possibility that GABA supplementation has great prospect in improving intestinal immunity and preventing intestinal infection through metabolism of intestinal microbiota in weanling piglets.

Therefore, this study is mainly aimed at examining the effects of dietary GABA supplementation on the growth performance, intestinal SIgA secretion, gut microbiota profiles, and metabolism in the normal and ETEC-infected weanling piglets. In total, we confirmed that the increased SIgA production is likely to be related to the activation of the T-cell-dependent pathway and altered intestinal microbial metabolism.

## 2. Materials and Methods

### 2.1. Bacterial Strain

An enterotoxigenic *Escherichia coli* F4-producing strain W25K (O149:K91, K88ac; LT, STb, EAST), which was isolated from a piglet with diarrhea [25], was used in the present study.

### 2.2. Piglets and Experiment Design

All procedures adopted in this experiment were approved by the Animal Welfare Committee of the Institute of Subtropical Agriculture, Chinese Academy of Sciences. A total of thirty-seven Duroc × Landrace × Yorkshire weanling piglets (5.82 ± 0.86 kg) were enrolled in the experiment at 21 d of age. The piglets were housed individually in an environmentally controlled nursery with hard plastic slatted flooring. All animals had free access to drinking water. The room temperature was maintained at 25 ± 2°C throughout the whole experiment. The composition and nutrient levels of the diets met the nutrient requirements for weanling piglets according to recommendations of the NRC (2012). The experiment lasted for three weeks and was divided into two stages. The first two weeks are the first stage, and the last week is the second stage. At the beginning of the experiment, all piglets were randomly distributed into two groups: (1) control group (CON, basal diet, *n* = 18) and (2) GABA group (GABA, basal diet with 40 mg·kg^−1^ of GABA supplementation, *n* = 19). On the 14th day of the experiment, 6 pigs in each group were slaughtered for sampling, and the remaining piglets were fed with ETEC to construct the infection model. Seven days after the modeling, 6 pigs were slaughtered for sampling in each group. According to whether or not ETEC was inoculated, it was divided into two stages: uninfected and infected (referred as CON1 and CON2 and GABA1 and GABA2). At the last day of every stage, six piglets from each group were randomly selected and sacrificed after anesthesia. Before being sacrificed, 10 mL blood was taken from the anterior vena cava and serum samples were obtained by centrifugation at 2000 × g for 10 min at 4°C and stored at -80°C. The liver, kidney, spleen, heart, and lung were obtained and weighed for calculating the relative weight of each organ. Samples from the same positions of the jejunum, ileum, colon, and feces were collected and immediately snap-frozen in liquid nitrogen and stored at −80°C for RNA extraction, determination of cytokine concentration, microbiota, and metabolite analysis.

### 2.3. Growth Performance and Organ Indices

Body weight and feed intake were recorded at the end of the first stage and the end of the whole experiment. The average daily gain and average daily feed intake were calculated with the ratio of total bodyweight gain to experimental days and the ratio of feed intake to experimental days. The feed conversion ratio is referred to as the ratio of the feed intake to the body weight gain.

### 2.4. Serum Amino Acid Analysis

Serum free amino acids were analyzed by high-performance liquid chromatography (HPLC) according to the manufacturer's instructions. The preprocessing of the samples was conducted as following description. In brief, firstly, 2 mL of serum samples was centrifuged at 3000 rpm for 5 minutes. Then 1 mL of supernatants was mixed with 0.8% sulfosalicylic acid solution. After being incubated at 4°C for 15 minutes, the mixtures were then centrifuged at 10,000 rpm for 10 minutes and filtered by a 0.22 *μ*m filter membrane before being analyzed by HPLC.

### 2.5. Serum Biochemical Analysis

Serum biochemical parameters were determined by the Biochemical Analytical Instrument (Beckman CX4) according to the instructions of manufacturer. And corresponding kits were bought from Roche (Shanghai, China).

### 2.6. Immunohistochemistry Analysis

The jejunum, ileum, and colon samples were fixed in 4% buffered paraformaldehyde for 24 hours at room temperature and embedded in paraffin and sectioned at a thickness of 3 mm. After being heated at 60°Cfor 30 to 60 minutes, the sections were dewaxed in xylene (10 min, twice) and then rehydrated in a descendent ethanol scale (100%, 95%, 85%, and 75%, 5 min every time). After being washed by distilled water for 5 minutes, the sections were soaked in a 0.01 M citrate buffer (pH = 6.0) and heated to boiling by a microwave oven for 25 minutes. When it had been cooled down and washed by PBS, 3% H_2_O_2_ was added into the buffer to inactivate the endogenous enzymes. The treated slides were incubated with primary antibodies (IgA, ab112746, Abcam), diluted at the ratio of 1 : 100, overnight at 4°C. Washed in PBS, the slides were incubated in 50~ 100 *μ*L biotinylated secondary antibodies and HRP-conjugated peroxidase at 37°C for 30 minutes. The chromogen was 3,39-diaminobenzidine free base (DAB).

### 2.7. ELISA

Equal amounts of samples were applied to examine IgA levels of the jejunum, ileum, and colon, as well as IFN-*γ*, IL-1*β*, IL-10, IL-13, IL-17, IL-4, and TNF-*α* levels of the jejunum by commercially available ELISA kits (Cusabio Biotech Company Limited, Wuhan, China) in accordance with the manufacturer's instructions.

### 2.8. Real-Time Quantitative PCR

Total RNA of ground jejunum tissue was isolated using the TRIzol reagent (Invitrogen, Carlsbad, CA, USA). The synthesis of complementary DNA was accomplished with the PrimeScript RT reagent kit with gDNA Eraser (Takara Bio Inc., Qingdao, China). RT-PCR was performed in duplicate with an ABI 7900 PCR system (ABI Biotechnology, MD, USA). Primers for the selected genes were designed using the Oligo 5.0 software (Molecular Biology Insights, Inc., USA) and Primer 6.0 software (PRIMER-e, New Zealand) and listed in Supplementary Table 1. *β*-Actin was used as an internal control to normalize target gene transcript levels. Relative expression of target genes was calculated by the 2^-*ΔΔ*Ct^ method [21]. The relative gene expression was expressed as a ratio of the expression of the GABA group to the controls.

### 2.9. Gut Microbiota Analysis

16S rRNA sequencing and general data analyses were performed by a commercial company (Novogene, Beijing, China). In brief, total genome DNA from samples was extracted using the CTAB/SDS method. The V3-V4 regions were amplified using the specific primer with the barcode. All PCR reactions were carried out in 30 *μ*L reactions with 15 *μ*L of Phusion® High-Fidelity PCR Master Mix (New England Biolabs), 0.2 *μ*M of the forward and reverse primers, and about 10 ng template DNA. Thermal cycling consisted of the initial denaturation at 98°C for 1 min, followed by 30 cycles of denaturation at 98°C for 10 s, annealing at 50°C for 30 s, and elongation at 72°C for 30 s, and finally 72°C for 5 min. PCR products were mixed in equidensity ratios. Then, mixture PCR products were purified with the GeneJET™ Gel Extraction Kit (Thermo Scientific). Sequencing libraries were generated using Ion Plus Fragment Library Kit 48 rxns (Thermo Scientific) following the manufacturer's recommendations. The library quality was assessed on the Qubit 2.0 Fluorometer (Thermo Scientific). At last, the library was sequenced on an Ion S5TM XL platform and 400 bp/600 bp single-end reads were generated. After the quality control, clean reads were obtained from single-end reads. Sequences analyses were performed by Uparse software (Uparse v7.0.1001). Sequences with ≥97% similarity were assigned to the same OTUs. Representative sequence for each OTU was screened for further annotation. Alpha indices (ACE, Chao1, observed species, Shannon, and Simpson) are applied in analyzing complexity of species diversity for a sample. Principal Coordinate Analysis (PCoA) was performed to get principal coordinates and visualize from complex, multidimensional data.

### 2.10. Metabolite Profiling Analysis

Untargeted metabolomics of piglet feces was performed by a commercial company (Novogene, Beijing, China). Preparation of samples could be briefly concluded as follows: dry completely in a vacuum concentrator without heating; add 60 *μ*L methoxyamination hydrochloride (20 mg/mL in pyridine) incubated for 30 min at 80°C; and add 80 *μ*L of the N,O-bis(trimethylsilyl)trifluoroacetamide reagent (1% trimethylchlorosilane, *v*/*v*) to the sample aliquots, incubated for 1.5 h at 70°C. All samples were analyzed by a gas chromatograph system coupled with a Pegasus HT time-of-flight mass spectrometer (GC-TOF-MS). GC-TOF-MS analysis was performed using an Agilent 7890 gas chromatograph system coupled with a Pegasus HT time-of-flight mass spectrometer. The system utilized a DB-5MS capillary column coated with 5% diphenyl cross-linked with 95% dimethylpolysiloxane (30 m × 250 *μ*m inner diameter, 0.25 *μ*m film thickness; J&W Scientific, Folsom, CA, USA). A 1 *μ*L aliquot of the analyte was injected in splitless mode. Helium was used as the carrier gas, the front inlet purge flow was 3 mL min^−1^, and the gas flow rate through the column was 1 mL min^−1^. The initial temperature was kept at 50°C for 1 min, then raised to 310°C at a rate of 20°C min^−1^, then kept for 6 min at 310°C. The injection, transfer line, and ion source temperatures were 280, 280, and 250°C, respectively. The energy was -70 eV in electron impact mode. The mass spectrometry data were acquired in full-scan mode with the *m*/*z* range of 50-500 at a rate of 12.5 spectra per second after a solvent delay of 4.78 min. Chroma TOF 4.3X software of LECO Corporation and LECO-Fiehn Rtx5 database were used for raw peak exacting, data baseline filtering and calibration of the baseline, peak alignment, deconvolution analysis, peak identification, and integration of the peak area. Both mass spectrum match and retention index match were considered in metabolite identification.

### 2.11. Microbiome-Metabolome Association Analysis

Pearson statistical method was used to calculate the correlation coefficients (rho) and *P* values of the contents of differential metabolites and relative abundances of differential bacteria. The absolute value of rho bigger than 0.6 and *P* value smaller than 0.05 were considered significant. Scatterplot and heat map of correlation analysis were drawn according to the results of Pearson statistical method.

### 2.12. Statistical Analyses

Data are shown as mean ± Standard Error of Mean (SEM). First, the D'Agostino-Pearson omnibus normality test (Prism 7.0) and Kolmogorov–Smirnov test (Prism 7.0) were applied to examine whether the data were in Gaussian distribution. If the data were in Gaussian distribution with equal variance, it would be analyzed by the unpaired *t*-test (Prism 7.0). If the data were in Gaussian distribution but with unequal variance, it would be analyzed by the unpaired *t*-test with Welch's correction (Prism 7.0). If the data were not in Gaussian distribution, it would be analyzed by the nonparametric test (Mann–Whitney *U* test, Prism 7.0). Differences with *P* < 0.05 were considered significant.

## 3. Results

### 3.1. GABA Supplementation Has No Effect on the Growth Performance and Organ Indices in Weanling Piglets

No significant difference was observed in the body weight gain, average daily gain, average daily feed intake, and feed conversion ratio between the CON1 and GABA1 groups (Fig. S1A). The growth performances of ETEC-infected piglets were not markedly influenced by GABA supplementation (Fig. S1B). In the first stage, organ indices had no difference between the CON1 and GABA1 groups (Table S2). From the perspective of the whole experiment, the organ indices of the GABA group were all higher than those of the CON group but without significant differences (Table S2).

### 3.2. GABA Supplementation Has Little Effect on the Serum Amino Acid Profile and Biochemical Indices

Compared with the CON1 group, the aspartic acid level in serum of the GABA1 group was significantly decreased (*P* < 0.05), while the histidine level in serum increased (*P* < 0.05) (Table 1). However, there was no difference in amino acid levels of serum between the CON2 and GABA2 groups (Table 1). As for the serum biochemical parameters, the calcium level in the serum of the GABA1 group was higher than that of the CON1 group (*P* < 0.05) (Table 2). For the ETEC-infected piglets, the albumin level in the serum was decreased in the GABA2 group compared with the CON2 group (*P* < 0.05) (Table 2).

### 3.3. GABA Supplementation Promotes Intestinal SIgA Production

**Figure 1:**
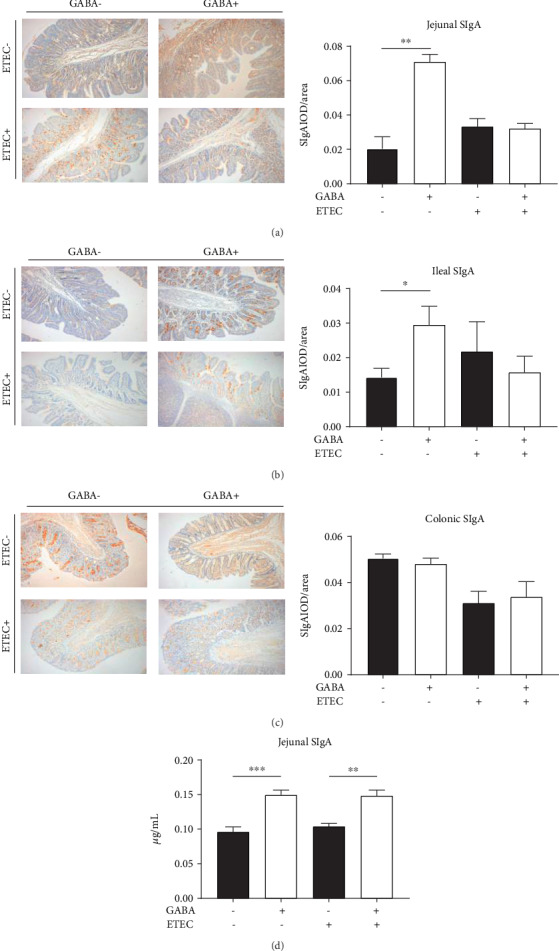
Dietary GABA promoted the production of SIgA in the intestine of piglets. Immunohistochemistry analysis of the IgA levels in the (a) jejunum, (b) ileum, and (c) colon of piglets in stage 1 and stage 2. (d) ELISA test of the jejunal SIgA level of piglets in stage 1 and stage 2. Data (*n* = 6) are presented as mean ± SEM and analyzed by the unpaired *t*-test with Welch's correction or Mann–Whitney *U* test. Differences were denoted as follows: ^∗^*P* < 0.05, ^∗∗^*P* < 0.01, and ^∗∗∗^*P* < 0.001.

To examine the effect of GABA supplementation on intestinal SIgA secretion, the jejunum, ileum, and colon were applied to the immunohistochemistry analysis. As the results show, GABA1 significantly improved the jejunal (*P* < 0.01) and ileal (*P* < 0.05) SIgA levels compared to CON1 (Figures 1(a) and 1(b)). However, GABA supplementation had no effect on the jejunal and ileal SIgA levels in ETEC-infected piglets (Figures 1(a) and 1(b)). With regard to the colonic SIgA level, neither healthy nor ETEC-infected piglets were affected by GABA supplementation (Figure 1(c)). Results from ELISA analysis further verified that GABA supplementation increased the jejunal SIgA secretion in both healthy piglets and infected piglets (*P* < 0.01) (Figure 1(d)).

### 3.4. GABA Supplementation Improves the Expression of SIgA-Related Cytokines, Especially in ETEC-Infected Piglets

**Figure 2:**
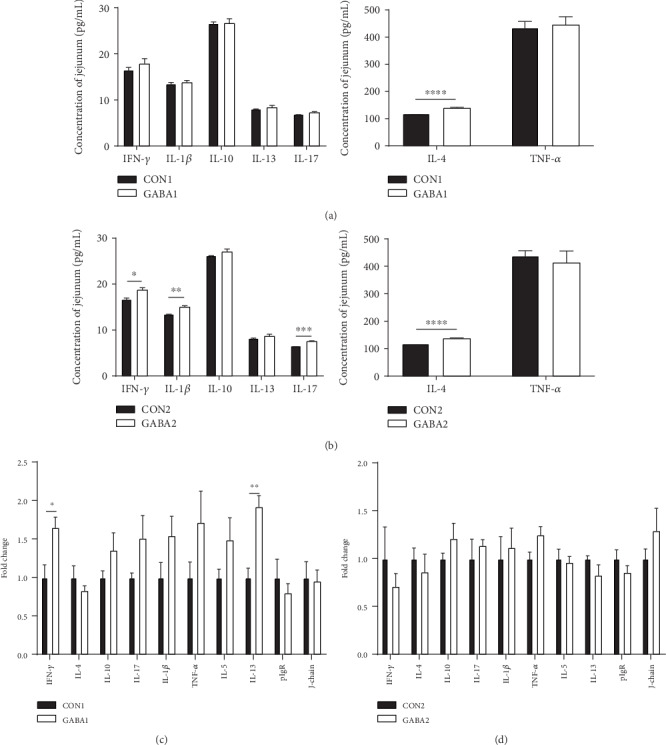
GABA promoted RNA expression and production of cytokines. (a) Jejunal concentrations of cytokines in normal piglets; (b) jejunal concentrations of cytokines in ETEC-infected piglets; (c) mRNA expression levels of cytokines in normal piglets; (d) mRNA expression levels of cytokines in ETEC-infected piglets. Data (*n* = 6) are presented as mean ± SEM and analyzed by the unpaired *t*-test with Welch's correction or Mann–Whitney *U* test. Differences were denoted as follows: ^∗^*P* < 0.05, ^∗∗^*P* < 0.01, ^∗∗∗^*P* < 0.001, and ^∗∗∗∗^*P* < 0.0001.

To explore the underlying mechanisms of the regulating effect of GABA supplementation on jejunal SIgA production, the protein levels and mRNA expression of cytokines in the jejunum of piglets were examined. The results of ELISA analysis showed that the jejunal concentration of IL-4 in the GABA1 group was much higher than that in the CON1 group (*P* < 0.0001) (Figure 2(a)). Compared with the CON2 group, GABA2 improved the jejunal concentrations of IL-4 (*P* < 0.0001), IFN-*γ* (*P* < 0.01), IL-1*β* (*P* < 0.01), and IL-17 (*P* < 0.001) (Figure 2(b)). Compared with the CON1 group, the mRNA expression of jejunal IFN-*γ* (*P* < 0.05) and IL-13 (*P* < 0.01) was significantly increased in the GABA1 group, while no significant alterations in the mRNA expression of any jejunal cytokines were observed in the GABA2 group (Figures 2(c) and 2(d)).

### 3.5. GABA Supplementation Alters the Relative Abundances of Gut Bacteria

**Figure 3:**
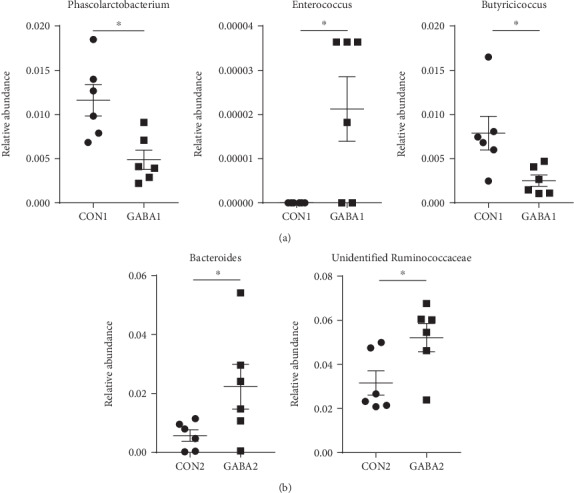
GABA altered the relative abundance of gut microbiota. (a) Relative abundance of *Phascolarctobacterium*, *Enterococcus*, and *Butyricicoccus* in normal piglets. (b) Relative abundance of *Bacteroides* and unidentified *Ruminococcaceae* in ETEC-infected piglets. Data are presented as mean ± SEM and analyzed by the unpaired *t*-test with Welch's correction or Mann–Whitney *U* test. Differences were denoted as follows: ^∗^*P* < 0.05.

To examine if GABA supplementation increased intestinal SIgA production through modulating gut microbiota, the fecal microbiota of piglets was analyzed by bacterial 16S rRNA sequencing (V3-V4 regions). An average of 85,463 raw reads was generated for each sample. After removing the low-quality sequences, 80,121 clean tags were clustered into OTUs for the following analysis, based on the 97% similarity level. As shown in Table S3, no matter in the healthy piglets or the ETEC-infected piglets, GABA supplementation had no effect on the diversity indices (Shannon and Simpson) or the richness indices (Chao1, ACE, and observed species). In addition, PCoA revealed that the gut microbiota composition of piglets basically was little affected by GABA treatment (Figs. S2F and S2L). The relative abundances of the top ten strains in the phylum, class, order, family, and genus level were not significantly altered by GABA supplementation (Figs. S2A to S2E). The same goes for the gut microbiota of ETEC-infected piglets (Figs. S2G to S2K). However, when the scope was not limited to the top ten strains anymore, there were some low-abundance strains that were markedly altered by GABA supplementation. In normal piglets, the relative abundances of *Phascolarctobacterium* (*P* < 0.05) and *Butyricicoccus* (*P* < 0.05) were decreased by GABA, while the relative abundance of *Enterococcus* (*P* < 0.05) was increased (Figure 3(a)). In ETEC-infected piglets, GABA supplementation increased the relative abundance of *Bacteroides* (*P* < 0.05) and an unidentified *Ruminococcaceae* (*P* < 0.05) (Figure 3(b)). These results suggested that GABA supplementation affected the relative abundances of certain low-abundance bacteria in weanling piglets.

### 3.6. GABA Supplementation Modulates Metabolism of Gut Microbiota, Especially in Normal Weanling Piglets

**Figure 4:**
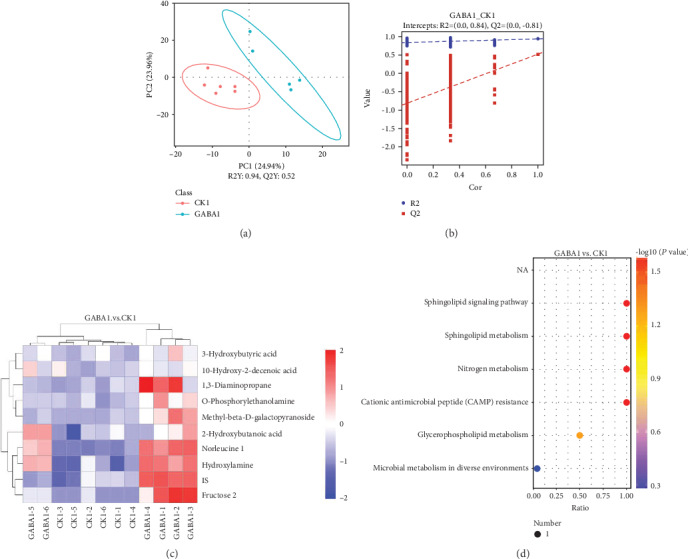
GABA highly changes the metabolism of gut microbiota in healthy piglets. (a) GABA1 vs. CON1 PLS-DA score plot; (b) GABA1 vs. CON1 PLS-DA valid plot; (c) heat map of significant variables; (d) scatterplot of KEGG enrichment analysis. The ratio in the scatterplot means the ratio of the number of altered metabolites to the number of all metabolites in this pathway. The CK group in this figure means the CON group.

The fecal metabolites were analyzed to examine whether GABA supplementation altered the metabolism of intestinal microbiota in weanling piglets. Firstly, a PLS-DA analysis was applied to have a better view of the different metabolic patterns in normal piglets. As shown in the PLS-DA score plot, the CON1 group and GABA1 group were distributed separately (Figure 4(a)). Quality of the resulting discriminant models suggested that the model was available and had good fitness and prediction (Figure 4(b)). Significant variables responsible for group separation were selected using the variable importance in the projection (VIP) statistic of the first principal component of the PLS-DA model (threshold > 1), together with the *P* value of Student's *t*-test (threshold < 0.05). As listed in Table 3, 10 metabolites were markedly upregulated in the GABA1 group. These metabolites were identified as potential markers: 2-hydroxybutanoic acid (*P* < 0.05), 1,3-diaminopropane (*P* < 0.05), IS (*P* < 0.05), O-phosphorylethanolamine (*P* < 0.05), methyl-beta-D-galactopyranoside (*P* < 0.05), 3-hydroxybutyric acid (*P* < 0.05), fructose 2 (*P* < 0.01), 10-hydroxy-2-decenoic acid (*P* < 0.05), norleucine 1 (*P* < 0.01), and hydroxylamine (*P* < 0.01) (Figure 4(c)). The results of KEGG enrichment analysis were presented as a scatterplot which showed that GABA markedly altered the sphingolipid signaling pathway, sphingolipid metabolism, nitrogen metabolism, cationic antimicrobial peptide (CAMP) resistance, glycerophospholipid metabolism, and microbial metabolism in diverse environments (Figure 4(d)).

**Figure 5:**
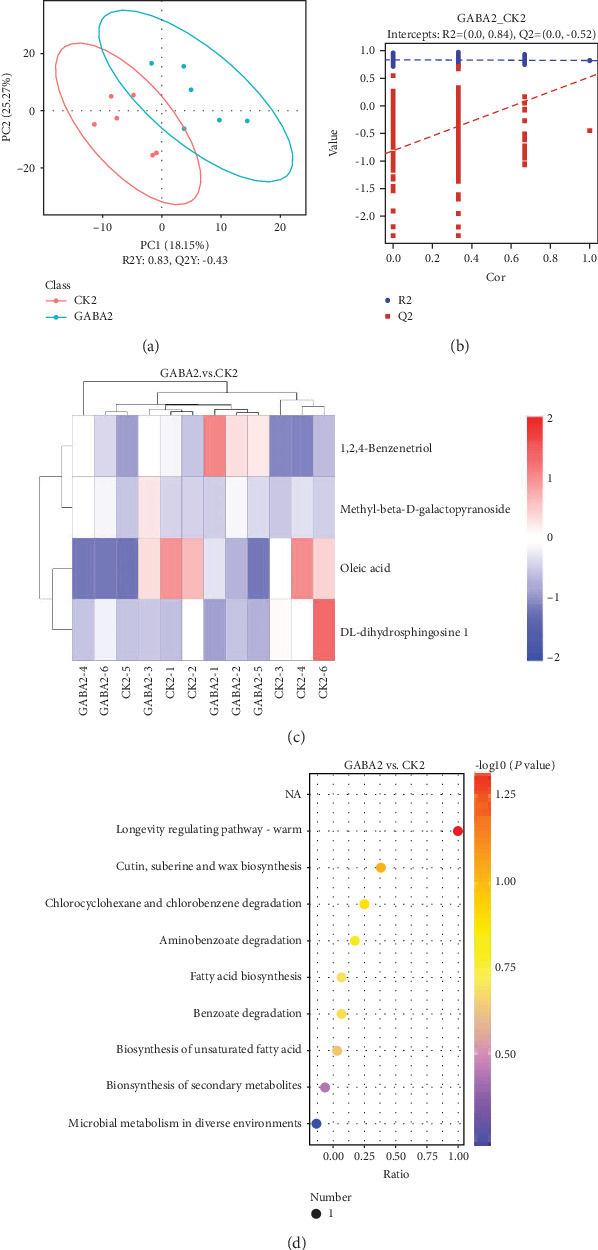
GABA regulated the metabolism of gut microbiota in ETEC-infected piglets. (a) GABA2 vs. CON2 PLS-DA score plot; (b) GABA2 vs. CON2 PLS-DA valid plot; (c) heat map of significant variables; (d) scatterplot of KEGG enrichment analysis. The ratio in the scatterplot means the ratio of the number of altered metabolites to the number of all metabolites in this pathway. The CK group in this figure means the CON group.

In the PLS-DA score plot, the GABA2 group and CON2 group were not separated from each other (Figure 5(a)). Only 4 metabolites were significantly altered: 1,2,4-benzenetriol (*P* < 0.01) and methyl-beta-D-galactopyranoside (*P* < 0.05) were upregulated, and oleic acid (*P* < 0.05) and DL-dihydrosphingosine 1 (*P* < 0.05) were downregulated (Figure 5(c)). The results of VIP statistic and fold changes are listed in Table 4. The scatterplot suggested that GABA significantly affected the longevity regulating pathway-worm in ETEC-infected piglets (Figure 5(d)).

We identified the specific metabolites related with enriched KEGG pathways and found that, in healthy piglets, all enriched KEGG pathways were related with O-phosphorylethanolamine or hydroxylamine (Table 5). In ETEC-infected piglets, two metabolites, oleic acid and 1,2,4-benzenetriol (Table 6), might mediate the effect of GABA supplementation on gut microbial metabolism in ETEC infection.

### 3.7. There Are Significant Correlations between Differential Metabolites and Altered Low-Abundance Bacteria

**Figure 6:**
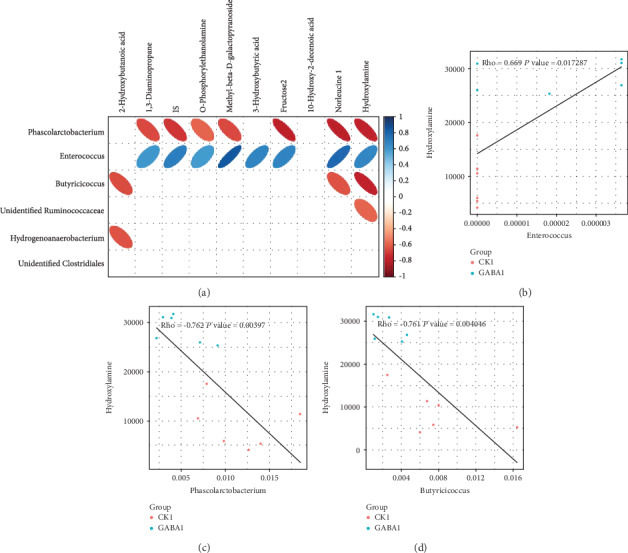
The relationship between gut microbiota and metabolites in healthy piglets. (a) Correlation analysis heat map of differential bacteria and differential metabolites in normal piglets; (b) correlation analysis scatterplot of the content of hydroxylamine and the abundance of *Enterococcus* in normal piglets; (c) correlation analysis scatterplot of the content of hydroxylamine and the abundance of *Phascolarctobacterium* in normal piglets. The CK group in this figure means the CON group.

**Figure 7:**
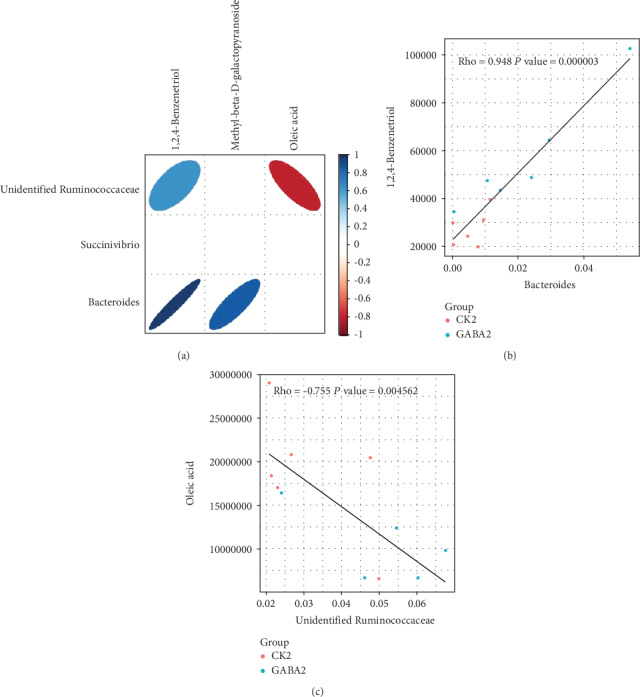
The relationship between gut microbiota and metabolites in ETEC-infected piglets. (a) Correlation analysis heat map of differential bacteria and differential metabolites in ETEC-infected piglets; (b) correlation analysis scatterplot of the content of 1,2,4-benzenetriol and the abundance of *Bacteroides* in ETEC-infected piglets; (c) correlation analysis scatterplot of the content of oleic acid and the abundance of unidentified *Ruminococcaceae* in ETEC-infected piglets. The CK group in this figure means the CON group.

We performed a microbiome-metabolome association analyses to examine the possible correlation between the altered low-abundance bacteria and the changed microbial metabolites. The results showed that hydroxylamine was positively correlated with *Enterococcus* (∣rho | >0.6) (Figure 6(b)) but negatively correlated with *Phascolarctobacterium* (∣rho | >0.6) (Figure 6(c)) and *Butyricicoccus* (∣rho | >0.6) in healthy piglets (Figure 6(d)). The absolute values of the correlation coefficient between other observed differential metabolites, O-phosphorylethanolamine, and *Enterococcus* and *Phascolarctobacterium* were lower than 0.6 (Figure 6(a)). In addition, we also found 1,2,4-benzenetriol in the colon was positively correlated with *Bacteroides* (Figures 7(a) and 7(b)), and oleic acid was negatively correlated with an unidentified *Ruminococcaceae* (Figures 7(a) and 7(c)).

## 4. Discussion

As a multifunctional neurotransmitter, GABA has received a lot of attention for its essential role in conducting neural signals, improving sleep quality, and alleviating hot stress [17, 26, 27]. Dietary GABA supplementation improves the growth performance, serum parameters, feed intake, and immune function of various animals, such as broilers, lambs, and cows [27–29]. However, consistent with our results, there is no direct evidence which revealed that GABA supplementation contributed to the growth of weaning piglets in previous studies [19, 21]. But previous studies and our results both demonstrated that GABA could modify intestine immunity and metabolism condition [21, 22]. In the present study, 40 mg·kg^−1^ of GABA supplementation decreased the aspartic acid level but increased the histidine level in the serum of normal piglets, while there are no significant changes in serum amino acid contents of ETEC-infected piglets. Aspartic acid can be metabolized to glutamate, which is the precursor of GABA, through transamination [30]. Glutamate could also degrade to histidine [31]. GABA supplementation does not affect the serum glutamate concentration maybe due to the inhibition of absorption of aspartic acid and the promotion of glutamate degradation to histidine in piglets. Surprisingly, GABA supplementation increases the calcium concentration of serum in normal piglets but decreases the albumin content of serum in ETEC-infected piglets. Serum albumin represents the main determinant of plasma oncotic pressure [32], and serum calcium is closely related to calcium and phosphorus metabolism. Therefore, GABA may affect the plasma oncotic pressure and calcium and phosphorus metabolism.

SIgA is a 400 kDa molecule composed of the secretory components, J-chain and dimeric IgA [33]. As a major component of the intestinal immune barrier, SIgA plays an important role in maintaining intestinal health by clearing pathogenic microorganisms and interacting with intestinal commensal microorganisms [5, 34, 35]. Furthermore, the host may discriminate symbionts from pathogens by recognizing the coating of commensal bacteria by SIgA [36]. Thus, enough SIgA secretion in the gut is essential for the intestinal homeostasis. However, piglets, especially weaning piglets, are unable to get maternal immunoglobulins and usually cannot secret enough SIgA because of their underdeveloped intestinal immune system [11]. Interestingly, in the current study, results of the ELISA and immunohistochemistry analyses confirmed that GABA supplementation increases the jejunal and ileal SIgA levels in normal piglets. Various Th2 cytokines, such as transforming growth factor- (TGF-) *β*1, interleukin- (IL-) 4, IL-5, IL-6, IL-10, and IL-13, can promote the immature B cells differentiated into IgA-secreting plasma cells [13]. To further verify our results about jejunal SIgA secretion and explore the underlying effects of GABA supplementation on cytokine production, we examined the SIgA-secreting cytokines in mRNA and protein levels. We found that jejunal concentrations of IFN-*γ*, IL-1*β*, IL-4, and IL-17 were upregulated by GABA treatment in ETEC-infected piglets. IL-1*β* mediates the host inflammatory response to prevent infection [37]. IFN-*γ* not only can activate macrophages to enhance phagocytosis of pathogenic bacteria [38] but also is implicated in the induction of pIgR synthesis and dimeric IgA binding [39]. Differentiation of B cells into plasma cells secreting IgA occurs upon interactions with T-cells in the lamina propria in an environment rich in IL-4 and other Th2 cytokines [39]. Moreover, IL-17 is an IgA-inducing cytokine that can increase pIgR expression and therefore the rate of SIgA secreted into the lumen [40]. In total, our study provided evidences that GABA enhances intestinal immunity by promoting SIgA secretion, and this might be the result of elevated levels of Th2 cytokines which further promotes the maturation of IgA-secreting plasma cells.

An extensive body of the literature has confirmed the interaction between SIgA and gut microbiota [13, 35, 41–43]. Interestingly, we detected several noteworthy strains altered by GABA supplementation. For example, GABA supplementation increased the relative abundance of *Enterococcus*, which has been widely proposed to be a probiotics that could be applied in porcine, murine, chicken, and even human models [44–47]. Feeding *Enterococcus faecium* significantly increases the intestinal SIgA level and promotes the proliferation of IgA^+^ cells in chicken and murine models [48–50]. Moreover, feeding dehydrated *Enterococcus faecium* increases the concentration of IL-4 in jejunal mucosa and decreases intestinal colonization of *Escherichia coli* in broilers [51]. Therefore, our results suggested GABA supplementation might enhance the SIgA secretion of intestine through increasing the relative abundance of *Enterococcus*.

The metabolite produced by gut microbes also exerts crucial roles in the health maintenance and modulation of physiologic function [52–56]. In the present study, we identified two metabolites, O-phosphorylethanolamine and hydroxylamine, which are significantly enriched by GABA supplementation in normal piglets. Interestingly, the content of hydroxylamine is positively correlated with the abundance of *Enterococcus* and associated with two enriched KEGG pathways: nitrogen metabolism and microbial metabolism in diverse environments. These results provided stronger implication that *Enterococcus* might play a key role in mediating the effect of GABA on intestinal SIgA secretion. On the other hand, the sphingolipid signaling pathway and sphingolipid metabolism are also enriched by GABA supplementation in normal piglets. Brown et al. demonstrated that sphingolipid produced by *Bacteroides* species can promote symbiosis with the host [57]. Meanwhile, the elevated abundance of *Bacteroides* in ETEC-infected piglets suggested that GABA supplementation might also affect SIgA secretion through *Bacteroides* and sphingolipid. However, the changes in microbial metabolism of ETEC-infected piglets are not as significant and organized as those in normal piglets. This could be attributed to the interference of ETEC infection, and further studies will be needed to clarify this issue.

In conclusion, although GABA supplementation had little effect on the growth performance, organ indices, serum amino acid profile, and serum biochemistry, it enhances intestinal immunity by promoting jejunal SIgA secretion. In addition, we observed interesting alterations in gut microbiota and microbial metabolism, which implies the potential mechanisms underlying the promotion of GABA supplementation on SIgA secretion. Our study provides insight into functional patterns of dietary supplementation on gut microbiome and host immune response and stresses the possibility of GABA utilization on intestinal health improvement.

## Figures and Tables

**Table 1:** Serum amino acid profiles of piglets in stages 1 and 2.

	CON1	GABA1	*P* value (1)	CON2	GABA2	*P* value (2)
Taurine	53 ± 6.62	47.09 ± 3.93	0.464	77.05 ± 12.9	84.51 ± 9.47	0.652
Aspartic acid	12.9 ± 12.9	7.11 ± 1.22^∗^	0.016	11.46 ± 2.91	11.11 ± 1.54	0.918
Threonine	27.82 ± 7.32	22.05 ± 5.6	0.547	28.68 ± 5.29	33.65 ± 7.71	0.608
Serine	48.89 ± 4.44	44.34 ± 2.2	0.388	46.72 ± 5.11	43.74 ± 7.44	0.749
Glutamic acid	119.99 ± 8.85	106.94 ± 12.61	0.419	130.03 ± 10.46	115.37 ± 10.49	0.346
Sarcosine	1.47 ± 0.43	1.77 ± 0.25	0.567	1.71 ± 0.45	1.75 ± 0.54	0.957
*α*-Aminoadipic acid	26.12 ± 3.03	26.13 ± 2.76	0.998	21.3 ± 4.17	22.79 ± 2.82	0.775
Glycine	368.99 ± 41.85	326.62 ± 28.04	0.423	234.47 ± 40.5	210.24 ± 66.01	0.762
Alanine	179.52 ± 18.95	143.21 ± 17.19	0.187	132.82 ± 19.87	124.6 ± 17.84	0.765
Citrulline	18.83 ± 0.78	22.88 ± 2.08	0.115	16.3 ± 1.4	14.79 ± 0.69	0.364
2-Aminobutanoic acid	4.12 ± 1.3	3.86 ± 0.73	0.866	10.94 ± 4.99	12.97 ± 3.13	0.738
Valine	57.92 ± 9.09	57.29 ± 5.27	0.954	72.64 ± 17.19	75.41 ± 7.8	0.888
Cysteine	11.97 ± 2.04	7.52 ± 1.16	0.095	12.47 ± 2.45	15.2 ± 3	0.498
Methionine	7.68 ± 0.87	7.57 ± 0.59	0.916	6.91 ± 0.78	7.29 ± 0.37	0.672
Cystathionine	7.75 ± 0.98	8.29 ± 1.09	0.720	6.01 ± 0.87	8.68 ± 1.24	0.112
Isoleucine	31.79 ± 3.12	33.52 ± 3.65	0.727	44.11 ± 9.79	54.03 ± 5.82	0.408
Leucine	59.29 ± 3.2	63.65 ± 2.31	0.298	61.87 ± 8.29	67.57 ± 3.78	0.551
Tyrosine	31.27 ± 3.21	36.63 ± 3.21	0.265	29.22 ± 2.03	26.57 ± 1.53	0.322
Phenylalanine	36.57 ± 2.39	38.35 ± 1.56	0.548	51.35 ± 8.64	56.15 ± 4.54	0.636
*β*-Alanine	7.82 ± 0.72	6.27 ± 0.5	0.112	6.38 ± 1.41	6.09 ± 1.04	0.874
3-Aminoisobutyric acid	0.26 ± 0.07	0.32 ± 0.09	0.597	0.23 ± 0.08	0.22 ± 0.06	0.915
*γ*-Aminobutyric acid	0.06 ± 0.02	0.05 ± 0.02	0.761	0.07 ± 0.02	0.04 ± 0.02	0.380
Ethanolamine	2.39 ± 0.25	2.22 ± 0.12	0.553	2.5 ± 0.19	2.68 ± 0.2	0.521
Hydroxylysine	1.21 ± 0.59	2.98 ± 0.77	0.099	1.16 ± 0.34	3.3 ± 1.61	0.245
Ornithine	31.15 ± 2.43	30.07 ± 1.74	0.726	32.15 ± 3.84	27.19 ± 1.59	0.273
Lysine	1.74 ± 11.02	93.03 ± 8.84	0.762	70.334 ± 7.35	78.54 ± 5.77	0.402
1-Methyl-L-histidine	6.01 ± 2.31	6.02 ± 1.74	1.000	4.77 ± 1.41	4.18 ± 1.41	0.773
Histidine	24.48 ± 1.99	32.26 ± 2.45^∗^	0.034	22.91 ± 2.95	23.62 ± 1.33	0.832
3-Methyl-L-histidine	4.3 ± 0.38	5.59 ± 0.46	0.059	5.68 ± 1.23	5.83 ± 0.48	0.915
Carnosine	10.51 ± 1.38	12.03 ± 0.96	0.389	6.01 ± 1.3	4.31 ± 0.89	0.307
Arginine	84.21 ± 7.29	87.61 ± 7.76	0.756	105.45 ± 17.44	106.34 ± 5.2	0.962
Proline	103.14 ± 5.08	95.3 ± 4.36	0.269	94.64 ± 12.63	84.46 ± 9.65	0.537

Means with ∗ are significantly different from the control group (*P* < 0.05).

**Table 2:** Serum biochemistry parameters of piglets in stages 1 and 2.

	CON1	GABA1	*P* value (1)	CON2	GABA2	*P* value (2)
Total protein	46.933 ± 0.750	45.8 ± 1.035	0.398	48.433 ± 1.410	47.267 ± 1.506	0.584
Albumin	37.75 ± 1.628	39.7167 ± 1.148	0.349	34.833 ± 1.114	31.200 ± 0.733^∗^	0.024
Alanine aminotransferase	31.85 ± 3.609	30.0833 ± 1.537	0.667	40.017 ± 8.316	43.217 ± 3.088	0.730
Aspartate aminotransferase	55.5 ± 9.549	53 ± 7.024	0.838	56.167 ± 10.058	51.500 ± 6.479	0.706
Alkaline phosphatase	309.833 ± 23.494	380.667 ± 49.876	0.239	199.333 ± 32.278	170.500 ± 20.343	0.470
Lactate dehydrogenase	555.167 ± 70.002	532.5 ± 25.160	0.770	612.667 ± 72.638	524.333 ± 46.760	0.335
Blood urea nitrogen	1.8333 ± 0.233	2.1333 ± 0.265	0.416	2.817 ± 1.009	2.617 ± 0.547	0.866
Glucose	5.6 ± 0.259	4.9667 ± 0.362	0.189	3.833 ± 0.549	4.467 ± 0.506	0.146
Ca	2.7017 ± 0.051	2.915 ± 0.070^∗^	0.036	2.632 ± 0.171	2.493 ± 0.082	0.490
P	2.208 ± 0.099	2.025 ± 0.191	0.420	2.868 ± 0.150	2.480 ± 0.107	0.064
Triglyceride	0.422 ± 0.030	0.405 ± 0.015	0.637	0.638 ± 0.178	0.845 ± 0.151	0.398
Cholesterol	1.821 ± 0.102	1.675 ± 0.119	0.373	2.127 ± 0.111	2.563 ± 0.202	0.096
High-density lipoprotein cholesterol	0.702 ± 0.054	0.580 ± 0.029	0.084	0.562 ± 0.099	0.520 ± 0.068	0.767
Low-density lipoprotein cholesterol	0.897 ± 0.099	0.832 ± 0.086	0.630	1.245 ± 0.112	1.648 ± 0.195	0.110
D-Lactic acid	8.967 ± 0.789	7.417 ± 0.320	0.114	9.763 ± 0.820	8.06 ± 0.683	0.143
Blood ammonia	172.567 ± 8.083	193.35 ± 6.764	0.078	299.367 ± 16.071	263.017 ± 5.825	0.075
Immunoglobulin M	0.067 ± 0.014	0.080 ± 0.012	0.481	0.172 ± 0.112	0.167 ± 0.090	0.973
Diamine oxidase	1.2 ± 0.157	1.433 ± 0.203	0.257	1.767 ± 0.275	1.500 ± 0.197	0.451

Means with ∗ are significantly different from the control group (*P* < 0.05).

**Table 3:** Significant variables responsible for group separation of the CON1 group and GABA1 group.

Metabolites	RT	VIP	*P* value	Fold change
2-Hydroxybutanoic acid	8.39152,0	1.0943912	0.0219111	2.08685768
1,3-Diaminopropane	15.2455,0	1.5156162	0.0389701	3.578994496
IS	16.6102,0	1.0352327	0.0446863	2.116515269
O-Phosphorylethanolamine	16.7466,0	1.1989025	0.0272898	2.718830077
Methyl-beta-D-galactopyranoside	17.4716,0	2.662989	0.0125204	8.738756131
3-Hydroxybutyric acid	8.87752,0	1.0999058	0.0356118	2.488895428
Fructose 2	17.7379,0	8.2363709	0.0037375	15.0103342
10-Hydroxy-2-decenoic acid	19.8381,0	1.1643369	0.0498178	2.53080591
Norleucine 1	11.1259,0	5.3345447	0.0008329	21.17542765
Hydroxylamine	8.2099,0	1.7659162	0.0021158	3.131884356

RT: retention time (min); VIP: variable importance in the projection of the PLS-DA first principal component; *P* value: *t*-test significance; fold change: GABA1 group to CON1 group.

**Table 4:** Significant variables responsible of the CON2 group and GABA2 group.

Metabolites	RT	VIP	*P* value	Fold change
1,2,4-Benzenetriol	14.717,0	1.331900147	0.005181206	2.074787321
Methyl-beta-D-galactopyranoside	17.4716,0	2.865360744	0.030532857	7.741120774
Oleic acid	21.1446,0	1.239038753	0.038381941	0.520647409
DL-dihydrosphingosine 1	23.2836,0	2.333778351	0.03935667	0.271034926

RT: retention time (min); VIP: variable importance in the projection of the PLS-DA first principal component; *P* value: *t*-test significance; fold change: GABA1 group to CON1 group.

**Table 5:** Enriched KEGG pathway and related differential metabolites in normal piglets.

Map ID	Map title	Metabolite
map00600	Sphingolipid metabolism	O-Phosphorylethanolamine
map00910	Nitrogen metabolism	Hydroxylamine
map01503	Cationic antimicrobial peptide (CAMP) resistance	O-Phosphorylethanolamine
map04071	Sphingolipid signaling pathway	O-Phosphorylethanolamine
map00564	Glycerophospholipid metabolism	O-Phosphorylethanolamine
map01120	Microbial metabolism in diverse environments	Hydroxylamine

Map ID: ID of the enriched KEGG pathway; map title: title of the enriched KEGG pathway; metabolite: specific differential metabolites related with the corresponding enriched KEGG pathway.

**Table 6:** Enriched KEGG pathway and related differential metabolites in ETEC-infected piglets.

Map ID	Map title	Metabolite
map04212	Longevity regulating pathway-worm	Oleic acid
map00073	Cutin, suberine, and wax biosynthesis	Oleic acid
map00361	Chlorocyclohexane and chlorobenzene degradation	1,2,4-Benzenetriol
map00627	Aminobenzoate degradation	1,2,4-Benzenetriol
map00061	Fatty acid biosynthesis	Oleic acid
map00362	Benzoate degradation	1,2,4-Benzenetriol
map01040	Biosynthesis of unsaturated fatty acids	Oleic acid
map01060	Biosynthesis of plant secondary metabolites	Oleic acid
map01120	Microbial metabolism in diverse environments	1,2,4-Benzenetriol

Map ID: ID of the enriched KEGG pathway; map title: title of the enriched KEGG pathway; metabolite: specific differential metabolites related with the corresponding enriched KEGG pathway.

## Data Availability

The data used to support the findings of this study are included within the article.

## Supplementary Materials

Supplementary Materials Supplementary Table 1: primers used in this study. Supplementary Table 2: organ indices of piglets in stages 1 and 2. Supplementary Table 3: gut microbial alpha diversity indices of piglets in stages 1 and 2. Figure S1: dietary GABA did not affect the growth performance of piglets. Growth performance of piglets in (A) stage 1 and (B) stage 2. Data are presented as mean ± SEM, analyzed by the unpaired *t*-test with Welch's correction or Mann–Whitney *U* test. Figure S2: dietary GABA had little effect on the composition of gut microbiota. (A–E) The relative abundances of main strains in the phylum, class, order, family, and genus level in normal piglets; (F) PCoA of gut microbiota in normal piglets; (G, H) the relative abundances of main strains in the phylum, class, order, family, and genus level in ETEC-infected piglets; (F) PCoA of gut microbiota in ETEC-infected piglets.
