# Supplementary material for: Effects of GABA Supplementation on Intestinal SIgA Secretion and Gut Microbiota in the Healthy and ETEC-Infected Weanling Piglets
Source: Mediators Inflamm. 2020 May 26;2020:7368483. doi: 10.1155/2020/7368483 (PMC7271228; doi:10.1155/2020/7368483)
Supplement: Supplementary Materials — Supplementary Table 1: primers used in this study. Supplementary Table 2: organ indices of piglets in stages 1 and 2. Supplementary Table 3: gut microbial alpha diversity indices of piglets in stages 1 and 2. Figure S1: dietary GABA did not affect the growth performance of piglets. Growth performance of piglets in (A) stage 1 and (B) stage 2. Data are presented as mean ± SEM, analyzed by the unpaired t-test with Welch's correction or Mann–Whitney U test. Figure S2: dietary GABA had little effect on the composition of gut microbiota. (A–E) The relative abundances of main strains in the phylum, class, order, family, and genus level in normal piglets; (F) PCoA of gut microbiota in normal piglets; (G, H) the relative abundances of main strains in the phylum, class, order, family, and genus level in ETEC-infected piglets; (F) PCoA of gut microbiota in ETEC-infected piglets. [file 7368483.f1.pdf]

Supplementary table 1. Primers used in this study.

Gene	Nucleotide sequence (5'-3')
IFN-γ-F	TTCAGCTTTGCGTGACTTTG
IFN-γ-R	GGTCCACCATTAGGTACATCTG
IL-4-F	CCCGAGTGTCAAGTGGCTTA
IL-4-R	TGATGATGCCGAAATAGCAG
IL-10-F	GGGCTATTTGTCCTGACTGC
IL-10-R	GGGCTCCCTAGTTTCTCTTCC
IL-17-F	ATCCTCGTCCCTGTCACTGC
IL-17-R	ACATGCTGAGGGAAGTTCTTGTC
IL-1β-F	CAGCCATGGCCATAGTACCT
IL-1β-R	CCACGATGACAGACACCATC
TNF-α-F	ACAGGCCAGCTCCCTCTTAT
TNF-α-R	CCTCGCCCTCCTGAATAAAT
IL-5-F	GACTGGTGGCAGAGACCTTGAC
IL-5-R	CTTCAATGCATAGTTGGTGATTTGT
IL-13-F	CTGACCACCAGCATGCAGTACT
IL-13-R	GCTGCAGTCGGAGATGTTGA
pIgR-F	AGCCAACCTCACCAACTTCC
pIgR-R	CTGCTAATGCCCAGACCAC
J-chain-F	GTGTGCCCGGATTACTTCCA

J-chain-R	AGTGGTGAGGTGGGATCAGA
β-actin-F	CTGCGGCATCCACGAAACT
β-actin-R	AGGGCCGTGATCTCCTTCTG

Supplementary table 2. Organ indices of piglets in stage 1 and 2.

	CON1	GABA1	*P* value (1)	CON2	GABA2	*P* value (2)
Heart	0.492±0.004	0.511±0.014	0.239	0.576±0.055	0.613±0.04	0.599
Liver	2.037±0.054	2.0±0.1	0.749	2.433±0.176	2.574±0.146	0.553
Spleen	0.159±0.015	0.155±0.017	0.870	0.198±0.019	0.23±0.024	0.309
Kidney	0.548±0.009	0.579±0.027	0.323	0.571±0.024	0.588±0.029	0.655
Lung	1.176±0.037	1.153±0.069	0.776	1.669±0.253	1.845±0.261	0.639

Supplementary table 3. Gut microbial alpha diversity indices of piglets in stage 1 and 2.

	CON1	GABA1	*P* value (1)	CON2	GABA2	*P* value (2)
Ace	562.68±41.39	519.47±22.77	0.382	606.04±9.31	627.72±22.43	0.393
Chao1	559.06±44.77	518.77±22.68	0.441	697.94±9.26	1015.20±316.82	0.34
Observed Species	519.67±35.85	483.50±27.65	0.443	565.00±11.57	562.17±24.96	0.92
Shannon	5.947±0.296	5.713±0.235	0.55	6.620±0.140	6.222±0.389	0.358
Simpson	0.941±0.016	0.946±0.010	0.834	0.974±0.003	0.939±0.027	0.214

Supplementary figure legends

Figure S1 Dietary GABA did not affect the growth performance of piglets. Growth performance of piglets in (A) stage 1 and (B) stage 2. Data are presented as mean ± SEM, analyzed by unpaired t-test with Welch's correction or Mann–Whitney *U* test.

Figure S2 Dietary had little effect on the composition of gut microbiota. (A) to (E) the relative abundances of main strains in the phylum, class, order, family, and genus level in normal piglets; (F) PCoA analysis of gut microbiota in normal piglets; (G) to (H) the relative abundances of main strains in the phylum, class, order, family, and genus level in ETEC infected piglets; (F) PCoA analysis of gut microbiota in ETEC infected piglets.

Figure S1

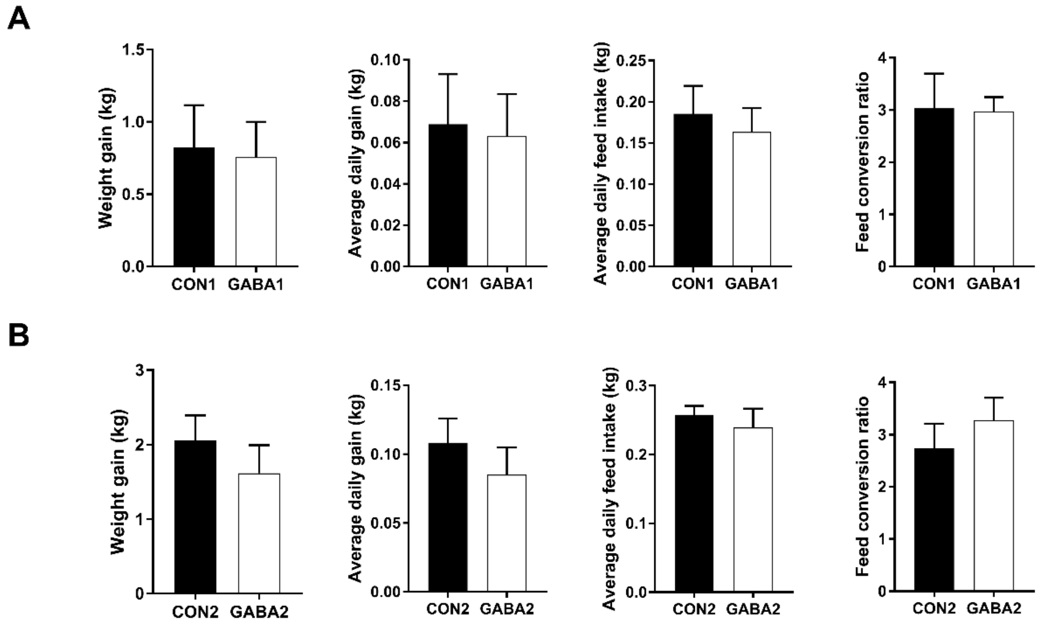
A
Weight gain (kg)
Average daily gain (kg)
Average daily feed intake (kg)
Feed conversion ratio
CON1
GABA1
B
Weight gain (kg)
Average daily gain (kg)
Average daily feed intake (kg)
Feed conversion ratio
CON2
GABA2

Figure S2

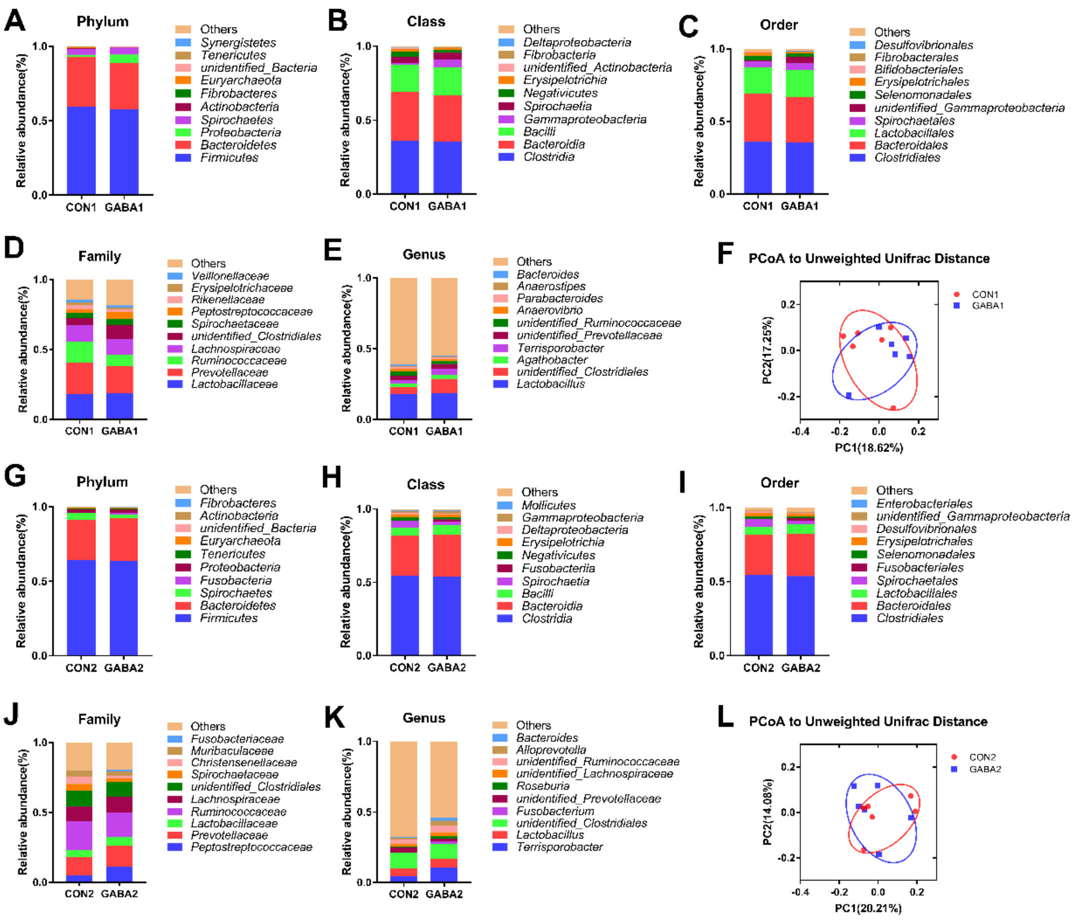
A
Phylum
Relative abundance(%)
CON1
GABA1
Others
Synergistetes
Tenericutes
unidentified_Bacteria
Euryarchaeota
Fibrobacteres
Actinobacteria
Spirochaetes
Proteobacteria
Bacteroidetes
Firmicutes
B
Class
Others
Deltaproteobacteria
Fibrobacteria
unidentified_Actinobacteria
Erysipelotrichia
Negativicutes
Spirochaetia
Gammaproteobacteria
Bacilli
Bacteroidia
Clostridia
C
Order
Others
Desulfovibrionales
Fibrobacterales
Bifidobacteriales
Erysipelotrichales
Selenomonadales
unidentified_Gammaproteobacteria
Spirochaetales
Lactobacillales
Bacteroidales
Clostridiales
D
Family
Others
Veillonellaceae
Erysipelotrichaceae
Rikenellaceae
Peptostreptococcaceae
Spirochaetaceae
unidentified_Clostridiales
Lachnospiraceae
Ruminococcaceae
Prevotellaceae
Lactobacillaceae
E
Genus
Others
Bacteroides
Anaerostipes
Parabacteroides
Anaerovibrio
unidentified_Ruminococcaceae
unidentified_Prevotellaceae
Terrisporobacter
Agathobacter
unidentified_Clostridiales
Lactobacillus
F
PCoA to Unweighted Unifrac Distance
CON1
GABA1
PC1(18.62%)
PC2(17.25%)
G
Phylum
CON2
GABA2
Others
Fibrobacteres
Actinobacteria
unidentified_Bacteria
Euryarchaeota
Tenericutes
Proteobacteria
Fusobacteria
Spirochaetes
Bacteroidetes
Firmicutes
H
Class
Others
Mollicutes
Gammaproteobacteria
Deltaproteobacteria
Erysipelotrichia
Negativicutes
Fusobacteriia
Spirochaetia
Bacilli
Bacteroidia
Clostridia
I
Order
Others
Enterobacteriales
unidentified_Gammaproteobacteria
Desulfovibrionales
Erysipelotrichales
Selenomonadales
Fusobacteriales
Spirochaetales
Lactobacillales
Bacteroidales
Clostridiales
J
Family
Others
Fusobacteriaceae
Muribaculaceae
Christensenellaceae
Spirochaetaceae
unidentified_Clostridiales
Lachnospiraceae
Ruminococcaceae
Lactobacillaceae
Prevotellaceae
Peptostreptococcaceae
K
Genus
Others
Bacteroides
Alloprevotella
unidentified_Ruminococcaceae
unidentified_Lachnospiraceae
Roseburia
unidentified_Prevotellaceae
Fusobacterium
unidentified_Clostridiales
Lactobacillus
Terrisporobacter
L
PCoA to Unweighted Unifrac Distance
CON2
GABA2
PC1(20.21%)
PC2(14.08%)
